# The impact of natural fibers’ characteristics on mechanical properties of the cement composites

**DOI:** 10.1038/s41598-022-25085-6

**Published:** 2022-11-29

**Authors:** Marzena Kurpińska, Magdalena Pawelska-Mazur, Yining Gu, Filip Kurpiński

**Affiliations:** 1 Gdansk University of Technology, Faculty of Civil and Environmental Engineering, Narutowicza 11/12, 80-233 Gdansk, Poland; 2 Suzhou Jcon Greenbuild Fabricated Design Co., Ltd., Suzhou, Jiangsu Province People’s Republic of China; 3 University of Gdańsk, Faculty of Law and Administration, Bażyńskiego 6, 80-309 Gdansk, Poland

**Keywords:** Ecology, Environmental sciences, Engineering, Materials science

## Abstract

The paper reviews the properties of cement composites reinforced with short fibres. The effect of natural fibres was investigated: cotton, sisal, jute, ramie, bamboo, and synthetic fibres: polymer and polypropylene. It was noticed that the fibres change the consistency of the mixture up to 15%. In the composite flexural strength tests, a change in strength by +/− 8% was observed, depending on the type of fibres used. The research shows that the use of natural fibres had a positive effect on the compressive strength by 27%, while the use of synthetic fibres caused its decrease by 4%. Additionally, it was noticed that the chemical composition, the diameter and the total length of the fibres in the element have an impact on the composite shrinkage. The fibre-containing composites showed an 8% higher water absorption compared to the non-fibre samples. The exception is the ramie fibres, which reduce water absorption. In general, a positive effect of natural fibers on the properties of cement composites has been noticed, however, in case of natural fibres application, a thorough further properties investigation is recommended.

## Introduction

The use of natural renewable materials becomes a good practice advised to reduce the carbon footprint^1^. Special attention is taken on materials made from plants sequestering CO_2_ from the atmosphere which can even provide a negative carbon footprint^2^.

Several researches were conducted on the use of different fibres as the reinforcement in concrete. It has been proven that the modification of the cement matrix by adding dispersed fibres reduces shrinkage and it has a positive impact on the cracking mechanics of mortars and concrete^3–6^. The traditional fibre reinforced concrete is composed of steel, polypropylene, glass, basalt or carbon fibres. However, the production of steel fibres or certain types of synthetic fibres is associated with a high energy consumption, high costs and environmental pollution^7–10^.

Compared with them, various natural plant fibres have the advantages of being cheap, recyclable, degradable, and renewable, with considerable strength and rigidity^11–14^. As their use may be limited, it is necessary to conduct research for using them in building construction^15–17^. Modification of physical and mechanical properties of mortars and concretes by natural fibres make the structure more resistant to load and shrinkage of elements^18–20^.

The percentage of dispersed natural fibre reinforcement usually is approx. 0.5–4% and this content of dispersed reinforcement allows for a significant modification of cement composites by changing the material into a quasi-elastic–plastic failure model^21–23^. It is possible, because the natural fibres are characterized by a modulus of elasticity from 5 to 130 GPa, and tensile strength from 200 to 1000 MPa. The use of natural fibres, however, requires a separate approach and scope of research in the field of their protection against water absorption, resistance to high temperatures and degradation, and ensuring the adequate adhesion to the cement matrix^24–27^.

Fibres such as sisal, jute, ramie, bamboo, kenaf, or cotton fibres were examed to determine the mechanical properties and suitability of using in cement composites^28,29^. In addition, the authors of this publication extended their research of which the main goal was to determine the possibility of using natural dispersed fibres as an alternative to synthetic fibres^30–33^. It was noticed that the use of natural fibres as reinforcement in cement composites has significant shortcomings. The incompatibility between the fibre and the cement matrix is one of them^34^.

Incompatibility between natural fibre and cement matrix can lead to low interfacial strength (ITZ) compared to polymer fibre. The main cause of this defect is the presence of hydroxyl groups and other polar groups in natural fibres, which makes them hydrophilic in nature. This hydrophilicity causes incompatibility with the hydrophobic cement matrix. The hydrophilicity of natural fibres indicates high fibre absorbability, which is the main cause of poor adhesion to the hydrophobic matrix. This phenomena in turn affects the friction and abrasion of the surface as well as the swelling or delamination of the fibres^35^.

The presence of moisture during the production of mortar or concrete mix may lead to poor workability and low mechanical properties of the composite. Although natural fibres are cheaper than their synthetic equivalent, in order to replace commonly used synthetic fibres, it is often necessary to improve their properties, e.g., by impregnating the surface with resins. These treatments impact the cost increase of the natural fibres. Nevertheless, it is worth looking for new applications for renewable natural fibres, so that they are not treated as the waste or used only as an alternative fuel.

It was decided to use natural fibres commonly available in various regions of the world (also of recycling different materials): bamboo, jute, cotton, ramie and sisal. These fibres are cheap and their use in cement composites can be economically justified.

### Modelling the stiffness of the cell wall of natural fibre

There is a strong correlation between the micro-fibril angle and the Young's modulus of the fibres. Models indicate that fibre stiffness is influenced by the spiral angle of the crystalline fibrils as well as the concentration of non-crystalline materials^36^.

**Figure 1:**
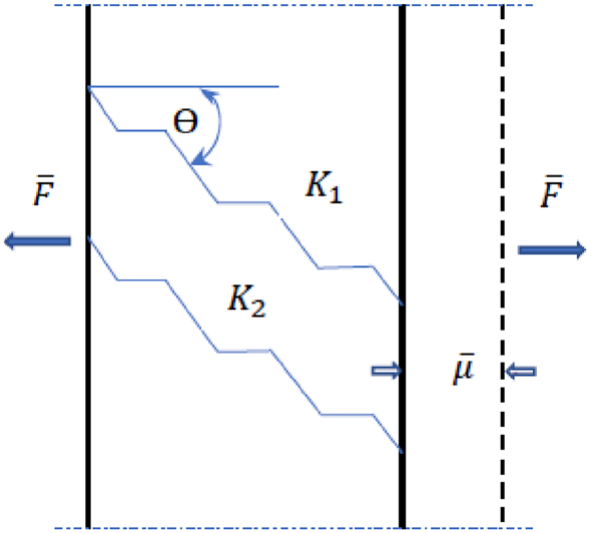
Elastic deformation model in the plant fibre cell wall.

These structural parameters vary between the different types of natural fibre, accounting for some of the variations in reported fibre properties. The effect on the mechanical properties of increased micro-fibril angle plays an important role when determining the mechanical properties of fibre-reinforced composites. It is necessary to measure the alignment of micro-fibrils applied to plant fibres to the direction of the force, especially in determining tensile properties, bearing in mind that plant fibres exhibit significant mechanical anisotropy. A theoretical analysis of the way a fibre behaves when stretched may in practice represent the behavior of fibre-reinforced composites when determining their mechanical properties. In this case a uniform strain theory has been used to obtain an estimate of the stiffness of the entire arrangement in both the fibre and the cell wall composite material. The theory uses an assembly of springs tied together so that they all receive the same displacement μ under a tensile load F, as shown in Fig. 1. The theory is based on the work of^37^.

The quantities $\overline{\mu }$ and $\overline{F }$ are the mean displacement and load on the micro-fibrils respectively ${K}_{1}$ and ${K}_{2}$ are the micro-fibril constants. F means the load and μ the displacement under tension. The total force necessary to produce the displacement is the sum of the forces in the fibre, F_f_, applied to the crystalline and amorphous regions (Eq. 1),

1 $${F}_{f}={F}_{C}+{F}_{nc}$$

where ${F}_{C}$ and ${F}_{nc}$ represent the forces applied to the crystalline and non-crystalline materials respectively. The stiffness (Eq. 2) of the array is given as:

2 $$k=\frac{F}{\mu }=\sum {K}_{i}$$

The components in Fig. 1 are at an angle *θ* to the direction of the force applied. A displacement μ is applied in the direction of the applied force $\overline{F }$. The forces, which appear in each spring element representation, are obtained from the product of the stiffness and the component of the displacement that is parallel to their orientation (Eq. 3).

3 $$\overline{{F }_{i}}={K}_{i}\overline{\mu }\mathit{cos}{\theta }_{i}$$

The total force in the longitudinal direction of the fibre, necessary to cause this displacement, is the sum of the longitudinal forces on each crystallite (Eq. 4).

4 $$\overline{F }={\sum F}_{i}\mathit{cos}{\theta }_{i}$$

Substituting ${F}_{i}$ from above gives Eq. (5).

5 $$\overline{F }=\overline{\mu }{\sum k}_{i}{\mathrm{cos}}^{2}{\theta }_{i}$$

The overall stiffness of the system of the fibre in the fibre axis is given by Eq. (6).

6 $$\overline{k }={\sum K}_{i}{\mathrm{cos}}^{2}{\theta }_{i}$$

If $\overline{k }$ overall stiffness of the fibre (${E}_{f}$) and ${K}_{i}$ is the stiffness of the micro-fibrils (${E}_{s}$) then Eq. (6) becomes (7).

7 $${E}_{f}={E}_{s}{\mathrm{cos}}^{2}{\theta }_{i}$$

Equation (7) gives the longitudinal Young’s modulus of plant fibres measured in tension.

## Materials and methods

The choice of mortar as a matrix in the research is mainly due to the following reasons:

- mortar is one of the most widely used materials in construction engineering. It is commonly used in bricklaying and plastering works.
- mortar test is simple and intuitive. Therefore, it is feasible to select the mortar as a research material that reflects the respective performance of concrete through the different properties of fibre-reinforced mortar. This subject is a quantitative study on the tensile, compressive properties and shrinkage of fibre-reinforced composites with different natural fibres but the same length and the same mass content.

The same requirements were given to the plain composite, polypropylene and polymer fiber-reinforced composite in order to compare their relevant performance.

### Materials

Tests were carried out on the standard composites modified with natural fibres and synthetic fibres. Research was mainly focused on the cement-based composites reinforced with natural fibres as the composites modified with synthetic fibres have already been thoroughly tested.

**Figure 2:**
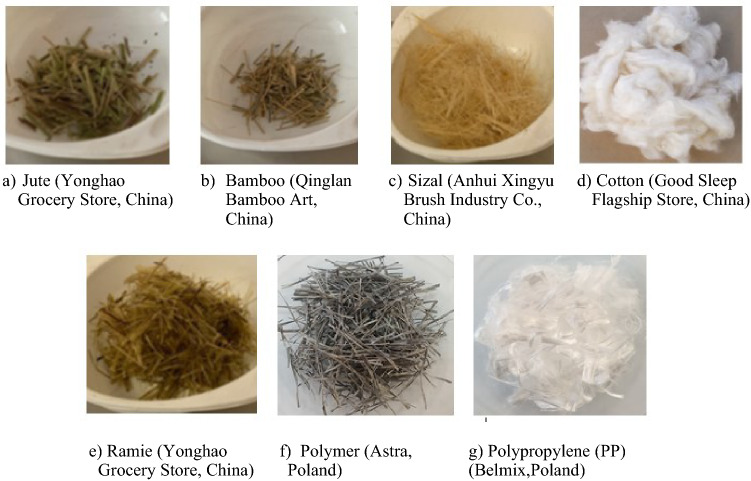
Fibre used in research [Fot. Y.Gu].

Composites with synthetic fibres as well as standard fibre-free composite were introduced only for the comparative samples**.** Five types of natural fibres: jute (Fig. 2a), bamboo (Fig. 2b), sisal (Fig. 2c), cotton (Fig. 2d), ramie (Fig. 2e) and two types of synthetic fibres: polymer-multifibre (Fig. 2f) and polypropylene (PP) (Fig. 2g) were used in research. The properties of natural and synthetic fibres are demonstrated in Table 1. The same requirement of the fibre length of 19 mm was given to all examined fibres.

**Table 1:** Properties of fibres used in research.

Fibers		PP	Polymer	Jute	Ramie	Sisal	Cotton	Bamboo
References		^4,23^	^11^	^5,12,24^	^5^	^4,20^	^14^	^12,33^
Length (l)	(mm)	19	19	19	19	19	19	19
Diameter (d)	(mm)	0.035	0.0375*	0.28	0.092	0.01	0.003	0.6
Slenderness (l/d)	(−)	543	507	68	210	190	6333	32
Number of fibres	(per 1 kg) (× 10^3^)	54,704	47,653	855	790	670,126	7,445,845	186
Total length	(m/kg) (× 10^3^)	1039	905	16	15	12,732	141,471	3.5
Total length of reinforcement (40 × 40 × 160 mm)	(m)	477,9	41,4	7,4	7	5856,7	65,076,7	1,6
Density	(g/cm^3^)	0.89–0.92	0.91–0.93	1.3–1.5	1.0–1.6	1.3–1.6	1.5–1.6	0.5–1.15
Young’s modulus	(GPa)	0.95–1.77	0.055–0.38	15–30	24.5–128	9–38	5.5–12.6	11–32
Tensile strength	(MPa)	26–41.4	40–78	320–800	400–1000	540–720	287–800	140–800
Elongation	(%)	15–700	90–800	1.0–1.9	3.6–3.8	2.0–3.3	3–10	2.5–3.7
Cellulose	(%)	–	–	59–70	70–83	65–76	80–94	40–45
Hemicellulose	(%)	–	–	15–20	10–17	10–16	–	25
Lignin	(%)	–	–	11–15	5–13	7–13	–	24
Natural moisture	(%)	0	0	2.5–5	8	5	0	0
Moisture absorption after 24 h	(%)	0,01–0,02	< 0.015	7–12	10–12	95–110	25–50	120–145

*^)^ was used multifilament polymer fibre 12-bundles with a total diameter of 0.01.

Cement: CEM II/B-V 42.5R Lafarge ordinary Portland (Cement Plant Kujawy, Poland) cement was used in research. The chemical composition and physical properties of cement are presented in Table 2. CEM II was used towards more sustainable composite which has a lower amount of Portland cement and therefore a lower carbon footprint. That cement is commonly used in fibre concrete for economic reasons. In addition, it has been already demonstrated that this cement shows a better behaviour in cementitious composites reinforced with natural fibres.

**Table 2:** Chemical composition and physical properties of CEM II/B-V 42.5R.

Setting start time (min)	Setting end time (min)	Compressive strength (MPa)			Blaine fineness (cm^2^/g)		Loss on ignition (%)		Water demand (%)
		2d		28d					
205	260	25.9		47.8	4411		4.1		28.5
**Content (%)**									
SiO_2_	Al_2_O_3_	Fe_2_O_3_	CaO	MgO	SO_3_	Na_2_O	K_2_O	TiO_2_	Cl^(-)^
25.1	8.8	3.2	54.0	1.2	2.4	0.19	0.67	0.18	0.059

**Figure 3:**
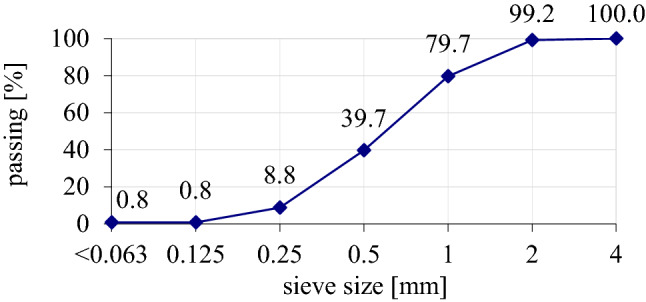
The properties of the grain size of fine aggregate 0–2 mm.

Sand from natural open aggregate pit was used as the aggregate, which was washed and sieved with a maximum diameter of 2 mm (Aggregate mine—Borowiec, Poland). The properties of the grain size of fine aggregate 0–2 mm were examined, and the results are presented on Fig. 3. Clean water from municipal water system was used to the mixture.

### Methods

**Fibre morphology** was determined using a scanning electron microscope (SEM, HITACHI Tokyo, Japan, version TM3030 Manual Stage, Model 55E-0015). The research was carried out in order to determine the surface structure and the fibre cross-section. Based on the microscopic examination of the fibre structure, the adhesion to the cement matrix was assessed. In total, 5 types of natural fibres and 2 types of synthetic fibres were tested.

Due to the special physical properties of seven fibres, the water-cement ratio selected in this experiment is 0.5. The mix ratio of composite selected in this experimental study is based on the mix ratio of ordinary composite, and the fibres in the fibre-reinforced composite are added in the form of admixture at the same amount of 1.8 kg/m^3^. The composition of test composite mix is shown in Table 3. The mixing ratio of composite: cement : sand : water is 1:3:0.5.

**Table 3:** Composition of composite mix.

Cement (kg/m^3^)	Water (kg/m^3^)	Sand (kg/m^3^)	Fibre (kg/m^3^)	Fibre (% c.c)
450	225	1350	1.8	0.4

The composite mix was prepared in an appropriate mixer (Automatic Mortar Mixers, AUTOMIX, Controls). At first, cement and water were dosed into the mix container, then the fibres were added together with sand. All ingredients were mixed for 2 min. Next, the fibre-composite was filled into three-part metal molds with the dimensions of 4 × 4 × 16 cm and vibrated. A total of 360 samples (45 samples of each type of fibre-composite) were done.

Composite **consistency tests** were carried out on a shock table designed to test the consistency of cement composites. The measure of consistency is the measurement of two perpendicular diameters in (mm) of the mixture flow of the mix-cement composites.

In order to assess the mechanical properties of the hardened fibre-composites, the research on the **flexural strength** and **compressive strength** was carried out. These tests were conducted on the samples with the dimensions of 4 × 4 × 16 cm, with the use of pressing machine manufactured by CONTROLS ADVANTEST 9 (Controls, Italy).

**Figure 4:**
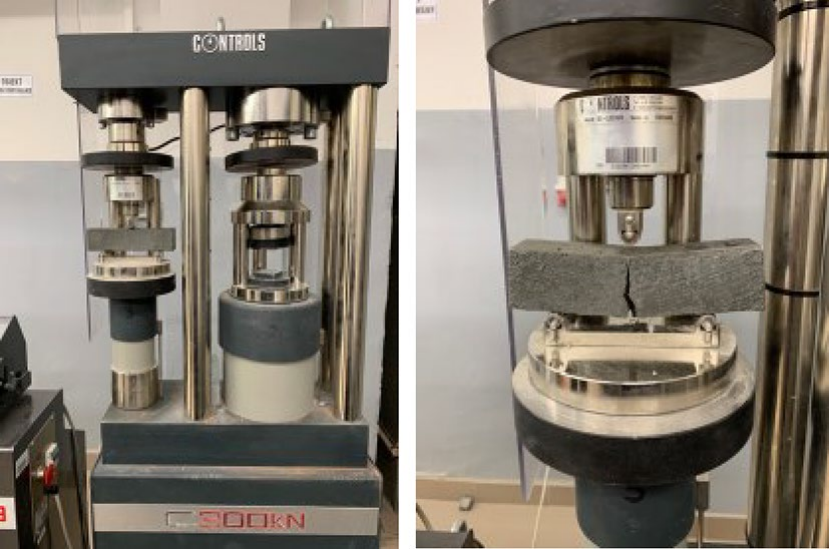
Flexural strength test [Fot.Y.Gu].

The flexural strength test was made on samples without notch in a 3-axis bending scheme. The bending force was applied at the centre of the sample span. The distance between the supports was 100 mm (Fig. 4).

The flexural strength of composite was calculated according to the formula (8):

8 $$\sigma =\frac{2FL}{3h{d}^{2}}$$

where: $\sigma$—flexural strength [MPa], $F$—load(force) at the fracture point [N], $L$—the length of the support span [mm,$h$—width of samples [mm], $d$—thickness of samples [mm].

Evaluation of the flexural strength of a group of three prisms is used as the test result.

**Figure 5:**
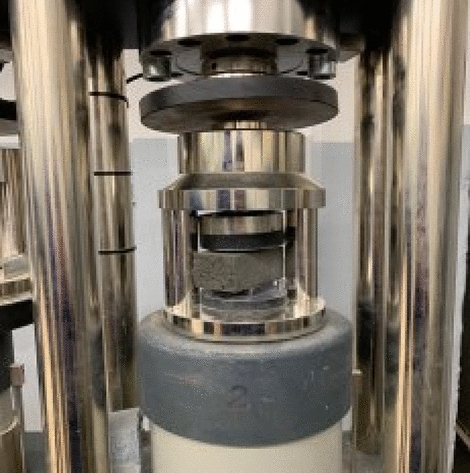
Compressive strength test [Fot. Y.Gu].

At the **compressive strength** test, the compression surface of the specimen was 40 × 40 mm (Fig. 5). Both tests were carried out in accordance with standard EN 197-1.

The compressive strength of concrete is calculated according to the formula (9):

9 $$\sigma =\frac{F}{{A}_{0}}$$

where: $\sigma$—compressive strength [MPa], $F$—load applied [N], ${A}_{0}$—area [mm^2^].

The **shrinkage rate** test was conducted with the use of the composite length comparator. The standard rod length is 160 mm, and the measurement accuracy is 0.01 mm. The shrinkage rate of concrete was calculated according to the formula (10):

10 $${S}_{n}=\frac{{l}_{1}-ln}{160}\cdot 1000$$

where: *S*_*n*_—drying shrinkage rate of composite in *n* days [mm/m], *l*_*1*_—length of composite sample on the first day [mm], *l*_*n*_—length of composite block on day *n* [mm].

All these tests were conducted after 1 day (*l*_*1*_), 2 days (n = 2), 3 days (n = 3), 7 days (n = 7), 28 days (n = 28), 56 days (n = 56), 90 days (n = 90) and 180 days (n = 180).

Each sample was tested immediately after it was removed from the mold to determine its initial length l_1_. The sample was then secured against drying by wrapping with foil. If l_1_ < l_n_ then the sample shows expansion, and if l_1_ > l_n_ the sample shows contraction.

The **water absorption** of composites samples tests was carried out on 3 samples with dimensions of 4 × 4 × 16 cm from every type of composite after 56 days of curing. The absorbability of the fibre-composite was conducted in the following steps: (a) drying samples at 105 °C to the constant weight; (b) gradual cooling of the samples for approx. 12 h; (c) determining the mass of the sample after drying, ${\mathbf{m}}_{0}$. The water temperature was constant at 20 ± 2 °C, (d) after 14 days of absorption the samples were taken out of the water and the surface was dried. The weight of the sample was identified with *m*s and the composite water absorption was calculated according to the formula (11):

11 $$\mathbf{n}=\frac{{\mathbf{m}}_{\mathbf{s}}-{\mathbf{m}}_{0}}{{\mathbf{m}}_{0}}\cdot 100\mathbf{\%}$$

where: *n*—water absorption of composite [%], *m*_s_—mass of test specimen after soaking [g], ${\mathbf{m}}_{0}$—mass of test specimen after drying [g].

## Results and discussion

### The structure and microstructure of the fibres

**Figure 6:**
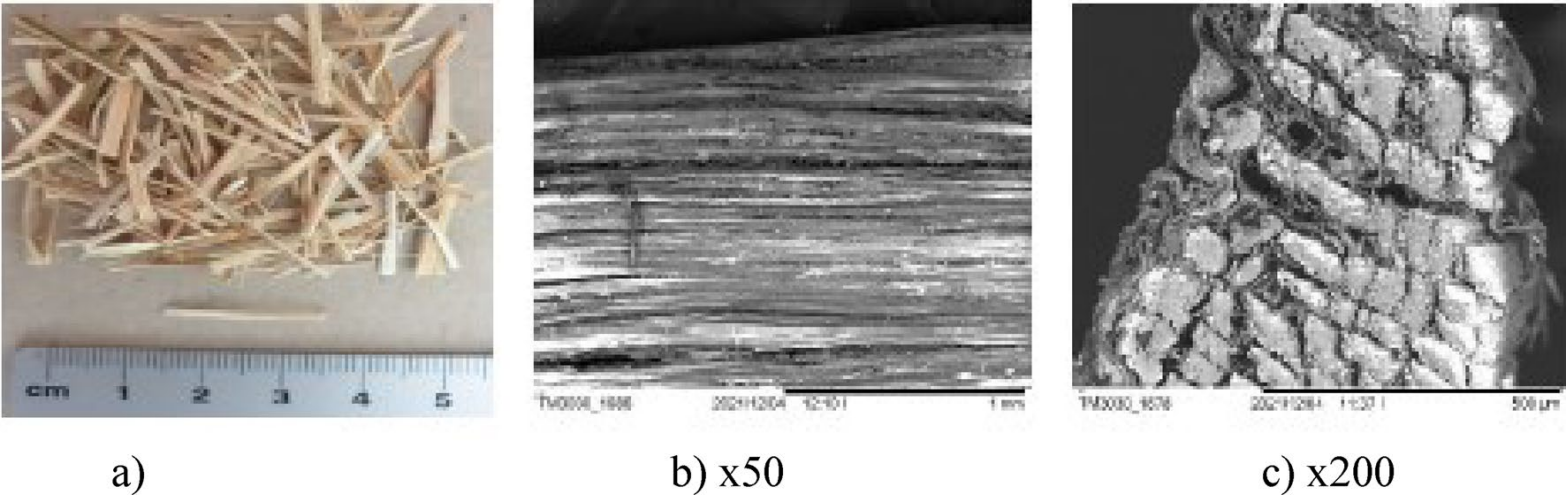
SEM of jute fibre [Fot.M.Kurpińska].

**Figure 7:**
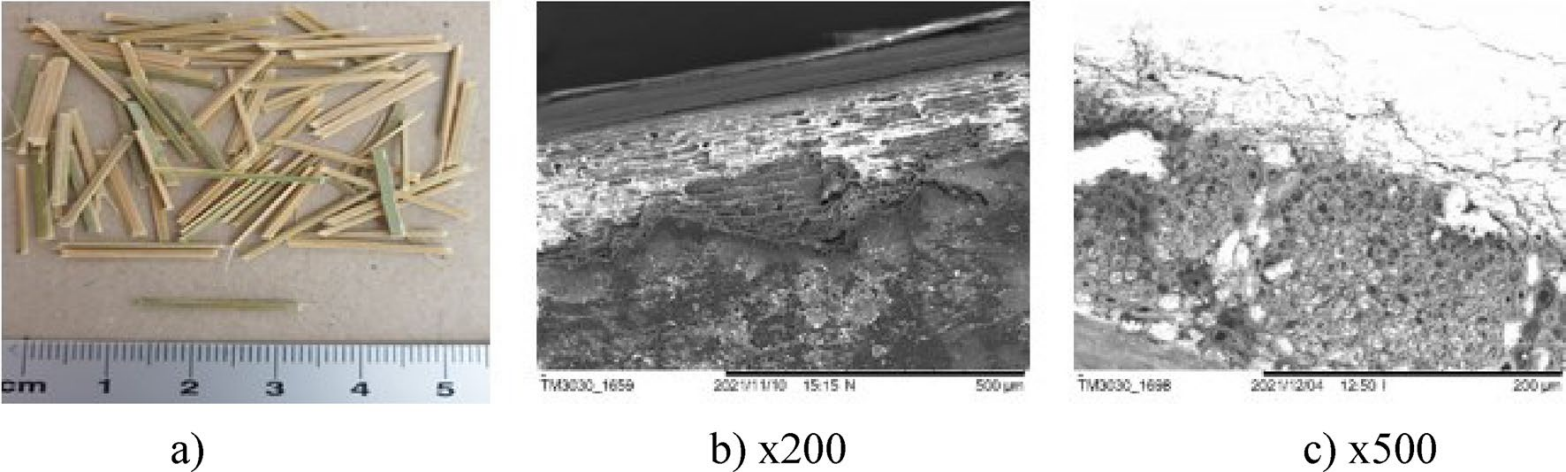
SEM of bamboo fibre [Fot.M.Kurpińska].

**Figure 8:**
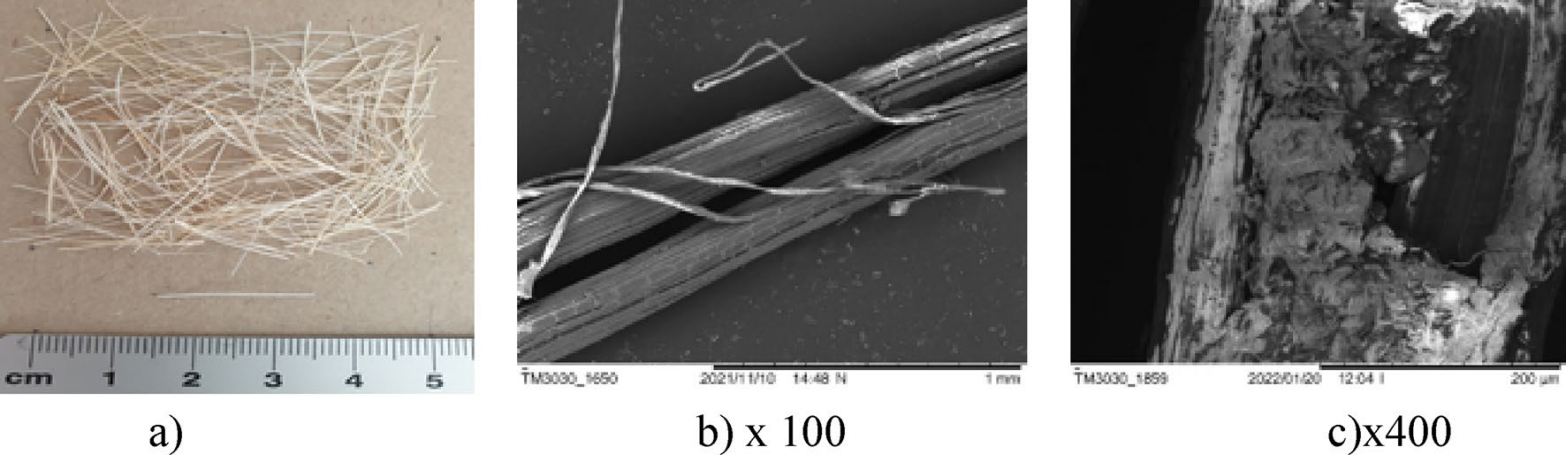
SEM of sisal fibre [Fot.M.Kurpińska].

**Figure 9:**
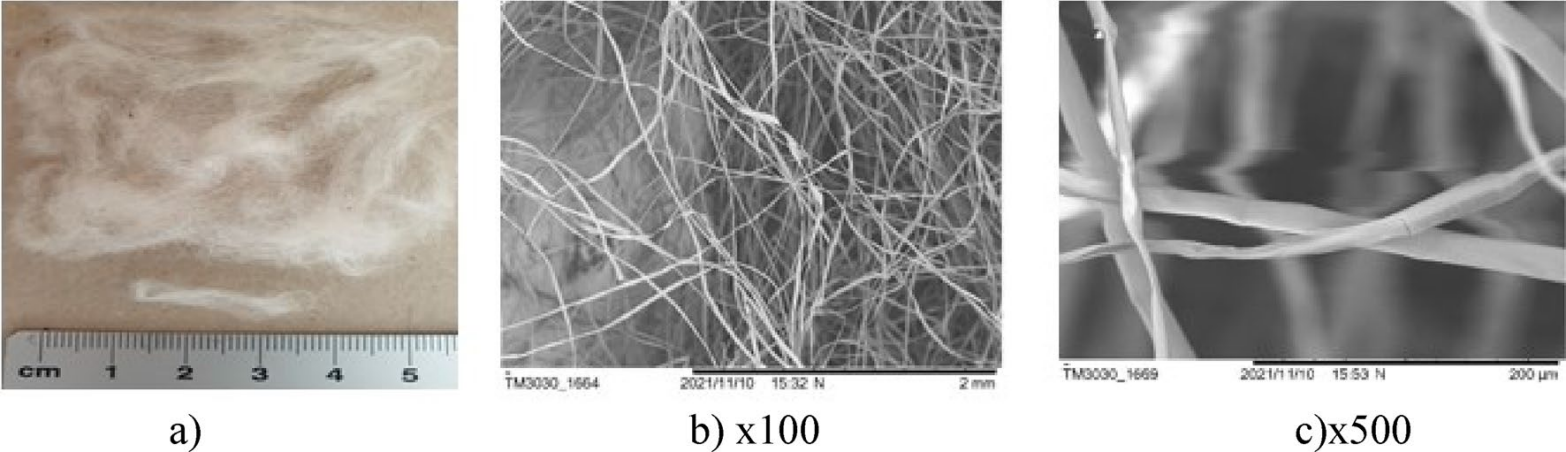
SEM of cotton fibre [Fot.M.Kurpińska].

**Figure 10:**
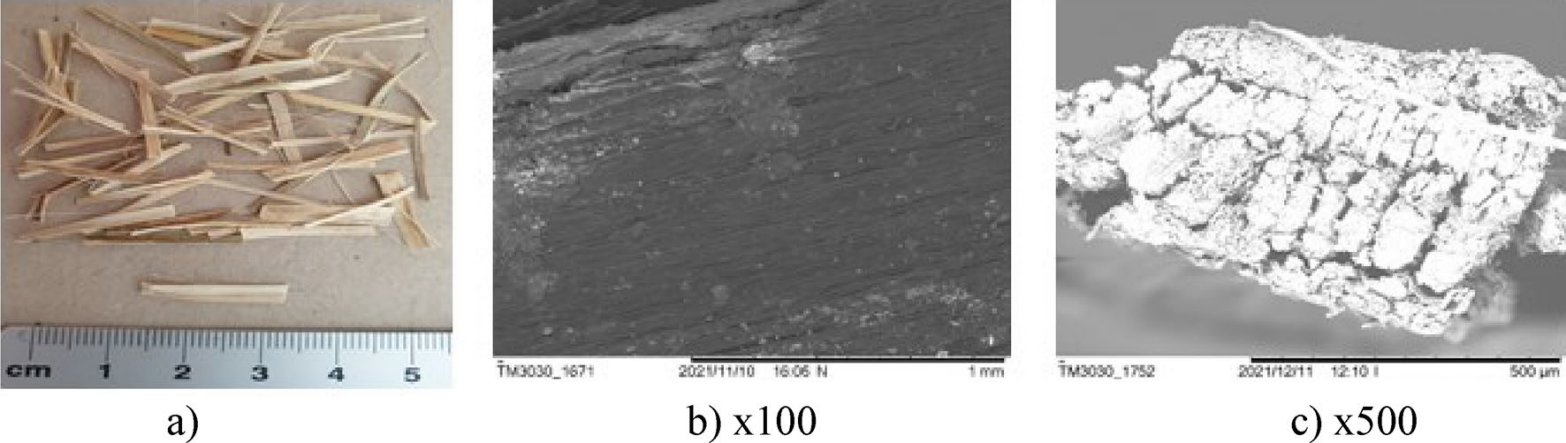
SEM of ramie fibre [Fot.M.Kurpińska].

**Figure 11:**
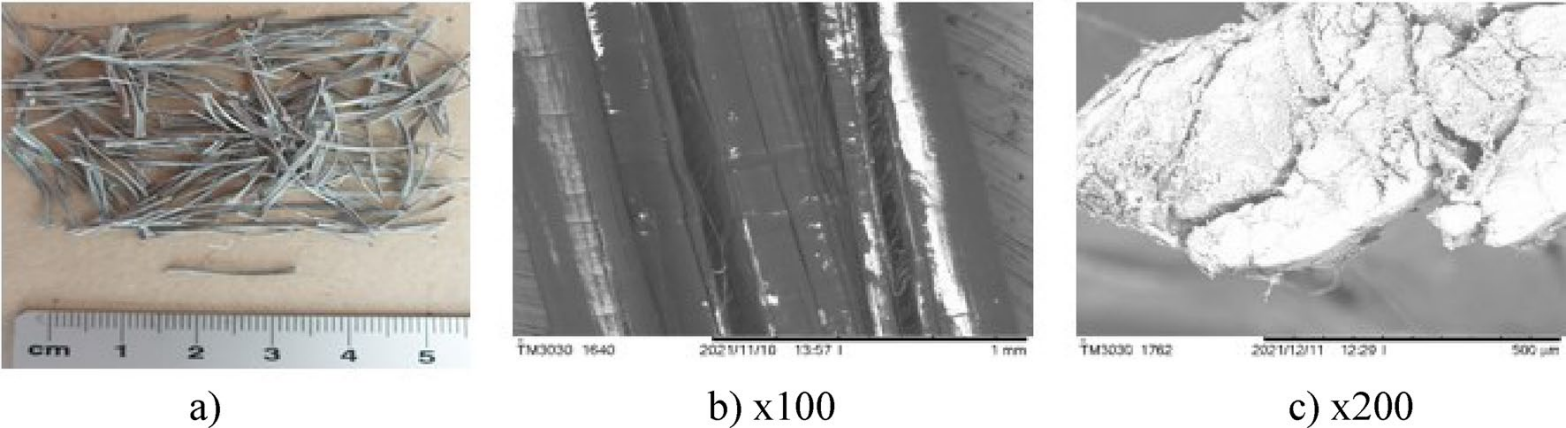
SEM of polymer fibre [Fot.M.Kurpińska].

**Figure 12:**
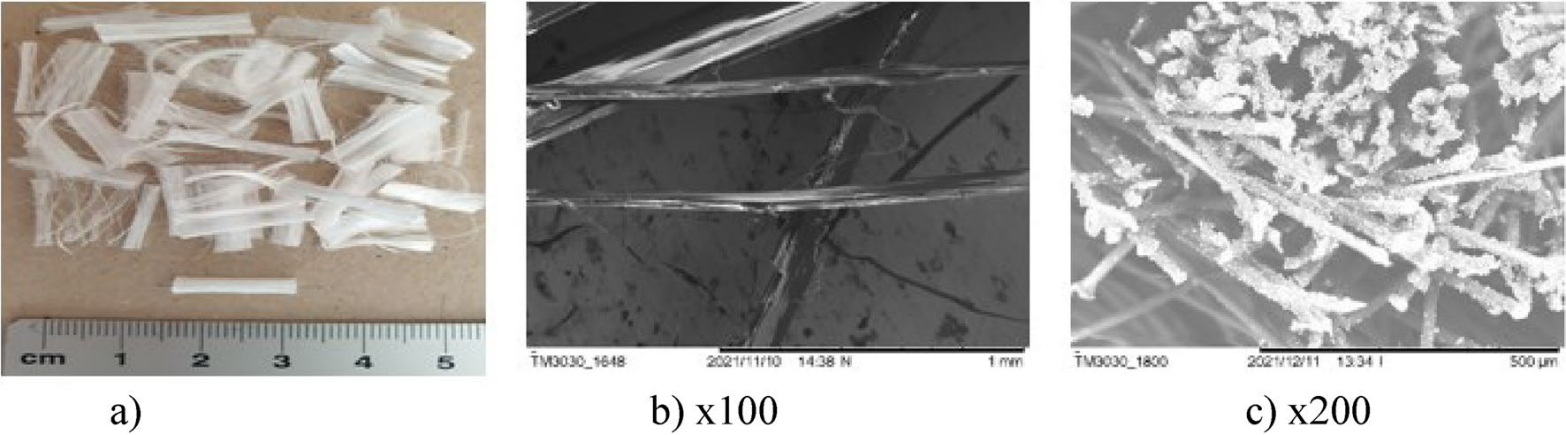
SEM of polypropylene (PP) fibre [Fot.M.Kurpińska].

The surfaces of the natural fibres are presented from Figs. 6, 7, 8, 9, 10 and of the synthetic fibres are presented in Figs. 11 and 12.

The basic components of natural fibres influencing their properties are cellulose, hemicellulose, lignin, waxes, oils, and pectin. Cellulose is mainly composed of three elements such as carbon, hydrogen, and oxygen, and it is the material basis that forms the cell wall natural fibre. Typically, cellulose remains in the form of micro-fibrils within the cell wall of a plant. Cellulose is the main factor affecting the tensile strength along natural fibre and the cellulose content is closely related to the plant's age and content decreases with the increasing age of the plant^6^.

Hemicellulose is an amorphous substance offering a low degree of polymerization and it exists between fibres. Hemicellulose is a complex polysaccharide with xylan as the predominant chain, and the branches mainly include 4-O-methyl-D-glucuronic acid, L-arabinose, and D-xylose. Lignin is a kind of polymer with complex structures and of many types. The basic units of lignin include: guaiacyl, syringyl monomers, and p-hydroxyphenyl monomers. The structural units in lignin are mainly connected by ether bonds and carbon–carbon single bonds. Usually, lignin is not evenly distributed in the plant fibre wall^9^.

In addition to three main components, lignin often contains various sugars, fats, protein substances, and a small amount of ash elements. These chemical compositions affect not only the properties of natural fibres, but also the possibility of a specific application of fibre. The composition of individual natural fibres and their properties are presented in Table 1. Figure 6a–c shows longitudinal and cross-sectional views of the untreated jute fibre. Externally, the fibre is smooth and shiny. The presence of hemicellulose influences the high hygroscopicity of jute fibres. The structure of the jute fibre shows that the fibre swells when it absorbs water. Possible swelling of the fibre in the cross-section by approx. 30%. The microscope scans of indicate the succinylated regions. This is due to the chemical bonding of the succinic anhydride molecule with the hydroxyl group of the cellulose present in the fibre. The encircled region in the top side shows an unsuccinylated region with naturally waxy impurities^16^.

Figure 7a shows the scanning electron micrograph (SEM) of the bamboo fibre. According to the SEM analysis, the microstructure of bamboo is anisotropic. At the Fig. 7b–c it can be recognized that the orientation of cellulose fibrils was placed almost along the fibre axis which may affect to maximize the modulus of elasticity. Factors affect the mechanical properties of bamboo fibres are the chemical composition and structure of bamboo fibres, moisture content, age of bamboo, etc. In addition, the age of the plant affects the chemical composition and structure of fibre. These factors and the natural humidity influence their change of mechanical properties. The hemicellulose content directly influences the tensile strength. This parameter increases with the decrease in the hemicellulose content in the bamboo fibre^18^.

The cell structure of bamboo fibres is complex, and the middle layer of the cell wall has a multi-layer structure. The lignification of the thin and thick layers in the multilayer structure varies. The multi-layered cell wall structure leads to better fracture resistance and promotes internal sliding between the cell wall layers during tension. The angle of the microfiber alignment is also an important factor influencing the mechanical properties of the fibre. Typically, the tensile strength and modulus of elasticity of a fibre increase as the angle between the interposition of the microfibers decreases. Hence, the smaller microfibril angle is an important factor that contributes to the good mechanical properties of bamboo fibre. Large voids between bamboo fibre molecules can be seen, which impact good hygroscopicity^19^. The moisture content is an important factor affecting the mechanical properties of bamboo fibres. Figure 8a–c shows the morphology of the sisal fibre. The surface of the sisal fibre has higher roughness, and it increases the bonding area between the fibre and cement paste. This leads to increase the mechanical properties of the composites^38^.

Figure 9a–c shows images of the cotton fibres. At the microscope image, a cotton fibre looks like a twisted ribbon or a collapsed and twisted tube. These twists are called convolutions: there are about 60 convolutions per centimetre. The weaves give the cotton an uneven surface of the fibres, which increases the friction between the fibres, but at the same time they can prevent fibres from evenly dispersing in the cement matrix. The outer layer, the cuticle is a thin film of mostly fats and waxes. Figure 9b shows the waxy layer surface with some smooth grooves. The waxy layer forms a thin sheet over the primary wall that forms grooves on the cotton surface^19^. The cotton fibre surface comprises non-cellulosic materials and amorphous cellulose in which the fibrils are arranged in a criss-cross pattern. Owing to the non-structured orientation of cellulose and non-cellulosic materials, the wall surface is unorganized and open. This gives flexibility to the fibre. The basic ingredients, responsible for the complicated interconnections in the primary wall, are cellulose, hemicelluloses, pectin, proteins, and ions. In the core of fibre, only the crystalline cellulose is present, what is highly ordered and has a compact structure with the cellulose fibrils lying parallel to one another^18^.

SEM micrograph of the surface and cross section of the ramie fibre are shown at Fig. 10a–c. The surface of the ramie fibres is dense but porous. There are many micropores and continuous bubbles in the porous structure of a single bundle of a ramie fibre Fig. 10c. This structure has some effect for low absorption of water, moreover, it is also related to the fibre distribution in the cement composites. In case of the short ramie fibre, due to its random distribution in composites, the strength of the composite may be affected. Cellulose, lignin, and hemicellulose weight materials can form a dense layer on the surface of the ramie fibres, so the water absorptivity is low. This special structure of the fibre with a dense matrix, and at the same time, with a characteristic pore arrangement has an influence on the adhesion of the cement matrix and the strength of the cement composite^18^.

The surface and cross section of multifilament macrofibre is demonstrated at Fig. 11a–c. From the chemical point of view, this type of fibres belongs to the polymers from the group of polyolefins, composed of units of the formula: –[CH_2_CH (CH_3_)]–. They are obtained by low-pressure polymerization of propylene. They are made of 100% pure co-polymer twisted bundles of multifilament fibres Fig. 11c. Polypropylene is one of two most commonly used plastics, in addition to polyethylene. Polypropylene is a hydrocarbon thermoplastic polymer^2^.

Figure 12a–c shows the structure of a bundle of polypropylene (PP) fibres in the form of a 3D mesh. They are made of isotactic polypropylene, called propylene, CH_2_=CHCH_3_ obtained from crude oil. They are one of the finest polypropylene fibres. The surface of the fibres is smooth Fig. 12b ^2^.

### The consistency—fluidity

**Figure 13:**
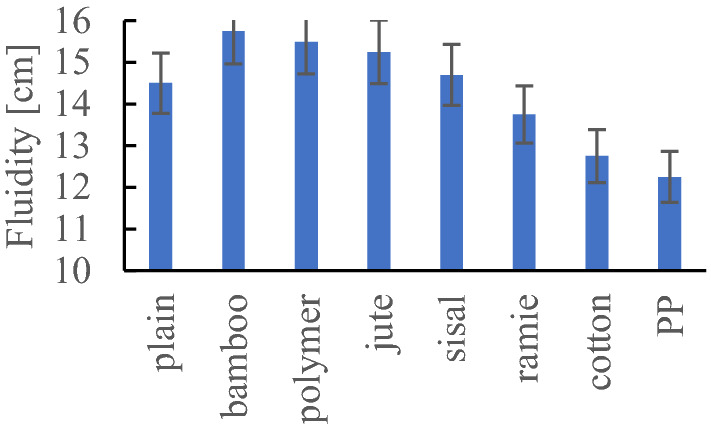
Results of fluidity test.

The results of fluidity are shown at Fig. 13. The fluidity of the composite not modified with fibres is 145 mm and is a reference to other test results. The use of bamboo fibres increased the composite fluidity and composite flow by 8.6% (157.5 mm). The use of polymer fibers and jute increased the consistency by about 7%, while the use of sisal fibres by 3%. The use of PP fibres (122.5 mm) had the greatest impact on the loss of consistency by 15.5%. The use of cotton and frame fibres resulted in a reduction of workability and consistency by 13.8% and 3.5%, respectively.

Based on the research results, it was found that in the case of using bamboo fibres characterizing a high absorption of 120–145%, the consistency of composite increased by 8.2% compared to the consistency of composite without fibres. In the case of a change in consistency, the chemical composition of natural fibres, their surface, and the total length in the volume of composite are significant, too. There is a noticeable regularity related to the cellulose content in natural fibres. If the higher cellulose content, it reduces the consistency of the composite. For example, the cellulose content in bamboo fibres is the lowest and amounts to 40–45%, while the cellulose content in cotton fibres is the highest, ranging from 80 to 94%. It can also be recognized that consistency and workability will be influenced by the hemicellulose content.

The higher the hemicellulose content, it impacts the higher consistency of the composite. It is similar referring to the content of lignin. It was noticed that the higher the lignin content, the higher the composite consistency was found. Regarding the total length of the fibres, a regularity is apparent that the greater the total length of fibres, e.g., in the case of cotton fibres, the greater decrease in consistency is visible. In the case of polymer and polypropylene (PP) fibres, the consistency is influenced by the surface of the fibre, the number of fibres, and their total length in the volume of the composite. Increasing the total length of PP fibres by approx. 15% resulted in a reduction of the consistency of approx. 20%.

### Flexural and compressive strength

**Figure 14:**
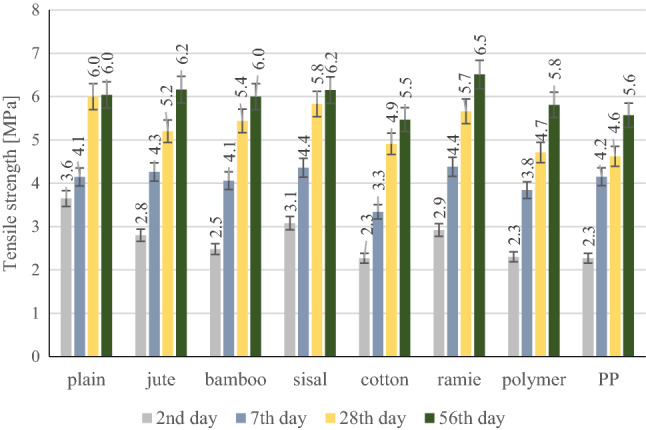
Flexural strength test results.

Assigning mechanical properties of fibre reinforced composite, particular emphasis was placed on the determination of the flexural strength of the composite. This parameter was appointed by the 3-point test. Figure 14. shows the flexural strength of plain composite and 7 groups of different fibre reinforced composites on the 2nd, 7th, 28th, and 56th days.

It can be seen that the bending strength of composites with the addition of natural fibres, ramie, bamboo, jute, and sisal are similar. The bending strength of composites with PP and polymer fibres is lower. It should be noted that the strength of the cotton fibre-reinforced composite is much lower than that of all the others tested. The reason may be the low tensile strength of the cotton fibres used. When mixing the composites, a tendency to create conglomerates of cotton fibres was also noticed, which may affect the strength of the composites.

**Figure 15:**
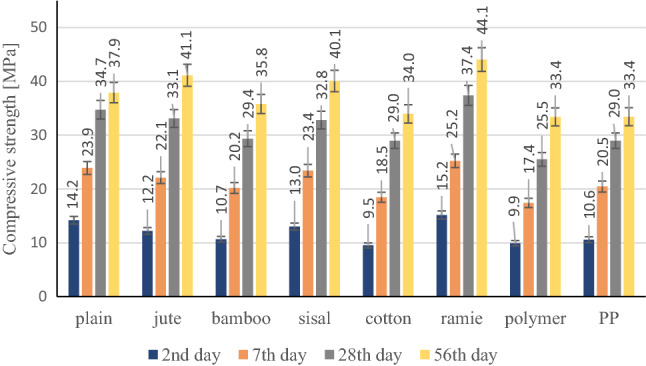
Compressive strength test results.

The test results clearly show that the effectiveness of the added natural fibres depends on the chemical composition and mechanical properties, and above all, on their adhesion to the cement matrix. The adhesion of the natural fibre to the cement matrix has a significant influence on the mechanical properties of the cement composite, in particular on compression and bending strength. The highest bending strength was achieved by cement composites modified with ramie fibres. Ramie fibres are characterized by the highest tensile strength among the tested synthetic and natural fibres, ranging from 400 to 1000 MPa. The results of the compressive strength are shown in Fig. 15.

The analysis of the test results shows that the use of dispersed fibres reduced the early compressive strength after 2 days from 8.5 to 33%. The exception is the ramie fibres, the use of which increased the early strength by 6.6%. Within 28 days, as in the case of early strength, the use of all types of synthetic and natural fibres resulted in a decrease in strength from 4.6 to 26.5%. The exception is the use of ramie fibres, which increased the compressive strength by 7.2% after 28 days. After 56 days, a decrease in strength was noticed in the case of using PP and polymer synthetic fibres as well as natural cotton and bamboo from 5.5 to 11.9%.

On the other hand, the increase in compressive strength after 56 days from 5.8 to 16.4% was visible in the case of using fibres such as sisal, jute and ramie. The highest compressive strength was achieved by the composite with a ramie fibre. The fibre of the ramie is characterized by the highest modulus of elasticity ranging from 24.5 to 128 GPa and is over 100% higher than the Young's modulus of the other fibres.

### Shrinkage test

**Figure 16:**
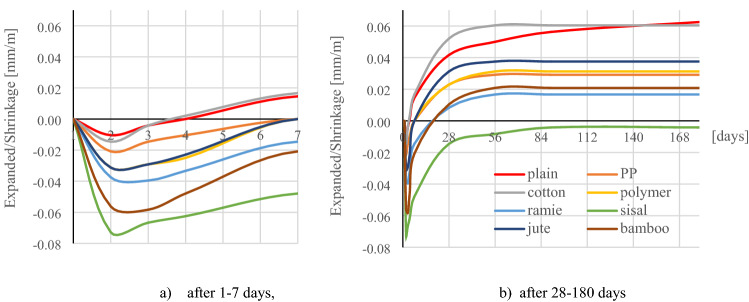
Testing the change in length of samples.

Figure 16A shows that the samples after demolding showed expansion for about 2 days, and from the third day after demolding, the length of the samples was shortened. The lowest degree of expansion in the first days was shown by samples without fibres and samples containing cotton fibres. In this case, the expansion did not exceed 0.02 mm/m. However, the same samples finally showed the highest shrinkage after 180 days, which was 0.06 mm/m.

The highest expansion within 48 h after deformation was shown by samples containing sisal fibres, while these samples finally after 180 days showed the lowest deformation of the length of the samples, which was 0.001 mm/m. The samples containing the synthetic fibres showed an expansion of about 0.02–0.03 mm/m in 48 h and the final shrinkage after 180 days was 0.03 mm/m for both the polymer and PP fibre samples. The bamboo and ramie fibres initially showed an expansion of 0.04–0.06 mm/m while their final shrinkage was 0.02 mm/m. The samples with jute fibres showed an expansion of 0.04 mm/m and the final shrinkage of the samples was 0.04 mm/m. Figure 16a,b shows the results of testing the change in length of samples over time.

After 180 days, the total deformation of the samples was determined. Samples containing sisal fibers showed a slight expansion of about 0.001 mm/m, while the highest deformation (shrinkage) was shown for composite samples without fibers and with cotton fibres, which was 0.06 mm/m. Samples with bamboo, jute, PP, polymer and ramie fibres showed a shrinkage from 0.02 to 0.04 mm/m. Only the samples containing the sisal fibre showed a slight expansion of 0.001 mm/m.

Ultimately, the samples containing sisal fibres were characterized by the lowest deformability. This phenomenon is related to the fibre structure and the total length of the fibres in a sample with dimensions of 40 × 40 × 160mm. For example, in a sample containing sisal fibres, their total length is 5856.7 m. Otherwise, a sample containing jute fibres, their total length in the sample is only 7.4 m. Therefore it was found that the fibre structure, its diameter, the cellulose content and the total length of the fibres in the element are important factors of deformation as a result of shrinkage or expansion of the fibre reinforced composite.

### Water absorption of composite test

**Figure 17:**
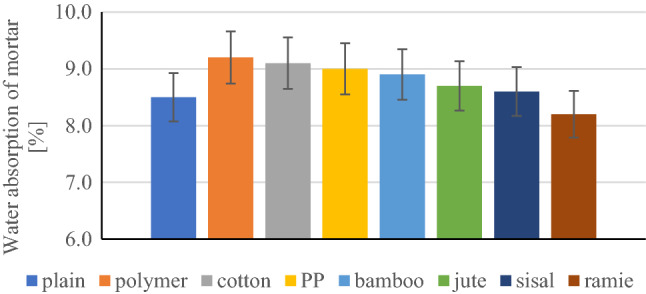
Water absorption of composite (%).

Higher water absorption (8.5%) compared to the composite without fibres was noticed in the case of using both synthetic fibres and with the exception of the use of ramie fibres, which caused a slight reduction in water absorption to 8.2%. It can be recognized that the water absorption rate of the 8 groups of samples is slightly different, the highest is the polymer fibre-reinforced composite (9.2%); the lowest water absorption rate refers to ramie fibre-reinforced composite (8.2%). The difference in water absorption rates is presented at Fig. 17.

Except for cotton fibre-reinforced composite, the water absorption rate of another plant fibre-reinforced composite is lower than that of synthetic fibre-reinforced composite. Probably because of the fact that ramie, sisal, and jute fibres all have good moisture absorption and release properties. It is commonly known that plant fibre-reinforced cement-based materials have reduced strength and initial properties due to their performance degradation in a humid environment, so their long-term durability could become problematic. Sisal fibres (with noticed absorption of 95–100%) have absorbed more cement slurry on their surface than jute fibres (absorption of fibre 7–12%). This phenomenon could be explained by the fact that the slurry became the impregnation of the fibre. The absorbability of the composite was tested after the composite had completely hardened. Probably a fibre that is characterized by high absorption—sisal is very well "embedded" in the matrix, therefore the bending strength results for composites with sisal fibre were higher by 8–10%.

## Future research

The results of the research and literature review show that natural fibre composites can be low-cost construction materials. Further tests should be performed in order to optimize the mechanical properties of the composites as well as to investigate other properties such as thermal conductivity and resistance for different environments.

The possible application for this kind of composite is the construction industry, so the planned research should involve frost resistance as well as high temperature (600–800 C) resistance and the durability issues. In the long perspective, it is recommended to research reinforcing lightweight concrete with optimal cement content and containing artificial lightweight aggregates.

## Conclusions

In general, during the conducted research, a positive effect of natural fibres on the properties of the composite was noticed. However, before using natural fibres, it is necessary to carefully recognize their properties.

During the study, it was noticed that natural and synthetic fibres change the rheology of the mixture. The bamboo fibres have changed the consistency to 10% more liquid, the ramie fibres did not change the consistency, and the synthetic fibres reduced the liquidity of the mixture by 15%.

During the tensile strength tests, almost no change in bending strength was noticed. The use of ramie fibres increased the tensile strength by 8%, while the use of cotton and PP fibres caused the decrease in tensile strength by 8%.

The research shows that the use of ramie fibres had a positive effect on the compressive strength of the samples and resulted in an increase in strength by 27%, while the use of synthetic fibres resulted in a decrease in compressive strength by 4%.

The use of fibres reduced the expansion of samples stored in water. Samples without fibres and samples with cotton fibres showed the greatest expansion. The smallest deformation was noted at sisal fibre samples. It has been observed that the expansion or shrinkage is influenced by the fibre structure, diameter, cellulose content and the total length of the fibres in the element.

The composites containing natural and synthetic fibres showed a higher water absorption of 2–8% compared to the absorption of the non-fibre samples. The exception is the ramie fibres, which reduced the water absorption by 3.5%.

Considering numerous advantages of natural plant fibres, such as light weight, low cost, easy availability, and recyclable decomposition, their application to composites reflects the increasing international requirements for sustainable concrete materials.

## Data Availability

The datasets used and/or analysed during the current study available from the corresponding author on reasonable request.

## References

[1] Zhong X, Hu M, Deetman S, Steubing B, Lin HX, Hernandez GA, Harpprecht C, Zhang C, Tukker A, Behrens P (2021). Global greenhouse gas emissions from residential and commercial building materials and mitigation strategies to 2060. Nat. Commun..

[2] Amran M, Fediuk R, Abdelgader H, Murali G, Ozbakkalogluf T, Lee YH, Lee YY (2021). Fiber-reinforced alkali-activated concrete: A review. J. Build. Eng..

[3] Azwa ZN, Yousif BF, Manalo AC, Karunasena W (2013). A review on the degradability of polymeric composites based on natural fibres. Mater. Des..

[4] Di Bella G, Fiore V, Galtieri G, Borsellino C, Valenza A (2014). Effects of natural fibres reinforcement in lime plasters (kenaf and sisal vs. Polypropylene). Constr. Build. Mater..

[5] Sanjay MR, Madhu P, Jawaid M, Senthamaraikannan P, Senthil S, Pradeep S (2018). Characterization and properties of natural fiber polymer composites: A comprehensive review. J. Clean. Prod..

[6] Vinod A, Sanjay MR, Suchart S, Jyotishkumar P (2020). Renewable and sustainable biobased materials: An assessment on biofibers, biofilms, biopolymers and biocomposites. J. Clean. Prod..

[7] Ramesh M, Deepa C, Kumar LR, Sanjay M, Siengchin S (2022). Life-cycle and environmental impact assessments on processing of plant fibres and its bio-composites: A critical review. J. Ind. Text..

[8] Jagadeesh P, Puttegowda M, Gowda Y, ThyavihalliGirijappa YG, Rangappa SM, Siengchin S (2022). Effect of natural filler materials on fiber reinforced hybrid polymer composites: An Overview. J. Nat. Fibers.

[9] Karimah A, Ridho MR, Munawar SS, Sudarwoko Adi D, Ratih Damayanti IR, Subiyanto B, Fatriasari W, Fudholi A (2021). A review on natural fibers for development of eco-friendly bio-composite: Characteristics, and utilizations. J. Mater. Res. Technol..

[10] Ku H, Wang H, Pattarachaiyakoop N, Trada M (2011). A review on the tensile properties of natural fiber reinforced polymer composites. Compos. Part B Eng..

[11] Mariak A, Kurpińska M (2018). The effect of macro polymer fibres length and content on the fibre reinforced concrete. MATEC Web Conf..

[12] Biswas S, Ahsan Q, Cenna A, Hasan M, Hassan A (2013). Physical and mechanical properties of jute, bamboo and coir natural fiber. Fibers Polym..

[13] Al-Zubaidi AB (2018). Effect of natural fibers on mechanical properties of green cement mortar. AIP Conf. Proc..

[14] Al-Ghaban A, Jaber H, Shaher A (2018). Investigation of addition different fibers on the performance of cement mortar. Eng. Technol. J..

[15] Han SO, Karevan M, Na Sim I, Bhuiyan MA, Hun Jang Y, Ghaffar J, Kalaitzidou K (2012). Understanding the reinforcing mechanisms in kenaf fiber/pla and kenaf fiber/pp composites: A comparative study. Int. J. Polym. Sci..

[16] Dinesh S, Sampath Kumaran P, Mohanamurugan S, Vijay R, Lenin Singaravelu D, Vinod A, Sanjay MR, Siengchin S (2019). Influence of wood dust fillers on the mechanical, thermal, water absorption and biodegradation characteristics of jute fiber epoxy composites. J. Polymer Res..

[17] Jenish I, Veeramalai Chinnasamy SG, Basavarajappa S, Indran S, Divya D, Liu Y, Sanjay MR, Siengchin S (2022). Tribo-Mechanical characterization of carbonized coconut shell micro particle reinforced with Cissus quadrangularis stem fiber/epoxy novel composite for structural application. J. Nat. Fibers.

[18] Kumar Setty V, Goud G, PeramanahalliChikkegowda S, MavinkereRangappa S, Siengchin S (2022). Characterization of chemically treated limonia acidissima (wood apple) shell powder: Physicochemical, thermal, and morphological properties. J. Nat. Fibers.

[19] Setty SN, Govardhan VK, MavinkereRangappa G, Siengchin S (2021). Raw and chemically treated bio-waste filled (Limonia acidissima shell powder) vinyl ester composites: Physical, mechanical, moisture absorption properties, and microstructure analysis. J. Vinyl Addit. Technol..

[20] Arpitha GR, Sanjay MR, Senthamarai Kannan P, Barile C, Yogesha B (2017). Hybridization effect of sisal/glass/epoxy/filler based woven fabric reinforced composites. Exp. Tech..

[21] Prakash R, Thenmozhi R, Raman SN, Subramanian C (2019). Fibre reinforced concrete containing waste coconut shell aggregate, fly ash and polypropylene fibre. Rev. Fac. Ing. Univ. Antioquia.

[22] Adediran AA, Akinwande AA, Balogun OA, Bello OS, Akinbowale MK, Adesina OS, Ojo AA (2022). Mechanical and optimization studies of polypropylene hybrid biocomposites. Sci. Rep..

[23] Yew MK, Mahmud HB, Ang BC, Yew MC (2015). Influence of different types of polypropylene fibre on the mechanical properties of high-strength oil palm shell lightweight concrete. Constr. Build. Mater..

[24] Rafiquzzaman M, Islam M, Rahman H, Talukdar S, Hasan N (2016). Mechanical property evaluation of glass–jute fiber reinforced polymer composites. Polym. Adv. Technol..

[25] Karahan O, Atiş CD (2011). The durability properties of polypropylene fiber reinforced fly ash concrete. Mater. Des..

[26] Onuaguluchi O, Banthia N (2016). Plant-based natural fibre reinforced cement composites: A review. Cem. Concr. Compos..

[27] Zakaria M, Ahmed M, Hoque MM, Hannan A (2015). Effect of jute yarn on the mechanical behavior of concrete composites. Springerplus.

[28] Hemath M, Mavinkere Rangappa S, Kushvaha V, Dhakal HN, Siengchin S (2020). A comprehensive review on mechanical, electromagnetic radiation shielding, and thermal conductivity of fibers/inorganic fillers reinforced hybrid polymer composites. Polymer Compos..

[29] Vinay SS, Sanjay MR, Siengchin S, Venkatesh CV (2021). Effect of Al_2_O_3_ nanofillers in basalt/epoxy composites: Mechanical and tribological properties. Polymer Compos..

[30] Ganapathy T, Sathiskumar R, Sanjay MR, Senthamaraikannan P, Saravanakumar SS, Parameswaranpillai J, Siengchin S (2021). Effect of graphene powder on banyan aerial root fibers reinforced epoxy composites. J. Nat. Fibers.

[31] Abhishek S, Sanjay MR, George R, Siengchin R, Parameswaranpillai J, Pruncu CJ (2018). Development of new hybrid Phoenix pusilla/carbon/fish bone filler reinforced polymer composites. J. Chin. Adv. Mater. Soc..

[32] Ganesan K, Kailasanathan C, Sanjay MR, Senthamaraikannan P, Saravanakumar SS (2020). A new assessment on mechanical properties of jute fiber mat with egg shell powder/nanoclay-reinforced polyester matrix composites. J. Nat. Fibers.

[33] Aweke HE, Mohammed TA (2020). The mechanical properties of fly ash concrete reinforced with bamboo fibers. Am. J. Constr. Build. Mater..

[34] Khalil HPSA, Tehrani MA, Davoudpour Y, Bhat AH, Jawaid M, Hassan A (2013). Natural fiber reinforced poly(vinyl chloride) composites: A review. J. Reinf. Plast. Compos..

[35] Mohanty AK, Misra M, Drzal LT (2001). Surface modifications of natural fibers and performance of the resulting biocomposites: An overview. Compos. Interfaces.

[36] Gassan J, Bledzki AK (1999). Possibility of improving the mechanical properties of jute/epoxy composites by alkali treatment of fibres. Compos. Sci. Technol..

[37] Bodig J, Jayne BA (1982). Mechanics of Wood Composites.

[38] Panthapulakkal S, Raghunanan L, Sain M, Tjong B, Baillie C, Jayasinghe R (2017). 4—Natural fiber and hybrid fiber thermoplastic composites: Advancements in lightweighting applications. Woodhead Publishing Series in Composites Science and Engineering, Green Composites.

